# High prevalence and clinical characteristics of respiratory infection by human rhinovirus in children from Lima-Peru during years 2009–2010

**DOI:** 10.1371/journal.pone.0271044

**Published:** 2022-07-15

**Authors:** Ariana Castañeda-Ribeyro, Johanna Martins-Luna, Eduardo Verne, Miguel Angel Aguila-Luis, Wilmer Silva-Caso, Claudia Ugarte, Hugo Carrillo-Ng, Angela Cornejo-Tapia, Yordi Tarazona-Castro, Juana del Valle-Mendoza

**Affiliations:** 1 School of Medicine, Research Center of the Faculty of Health Sciences, Universidad Peruana de Ciencias Aplicadas, Lima, Peru; 2 Laboratory of Molecular Biology, Instituto de Investigación Nutricional, Lima, Peru; 3 School of Medicine Alberto Hurtado, Universidad Peruana Cayetano Heredia, Lima, Peru; 4 Pediatrics Service, Hospital Nacional Cayetano Heredia, Lima, Peru; Defense Threat Reduction Agency, UNITED STATES

## Abstract

**Introduction:**

Human rhinovirus is a major cause of acute respiratory infections (ARIs) worldwide. Epidemiological data on human rhinovirus (RV) in Peru is still scarce, as well as its role in respiratory infections in children. Therefore, the aim of this study was to describe the prevalence of rhinovirus and to identify the circulating species in nasopharyngeal swabs from children with acute respiratory infections.

**Materials and methods:**

We analyzed nasopharyngeal swab samples that were collected from children younger than 17 years old, who had a clinical diagnosis of ARI from the “Hospital Nacional Cayetano Heredia” between May 2009 and December 2010. The original study recruited 767 inpatients with ARI, 559 samples of which were included and analyzed in the current study. Detection of rhinovirus and determination of rhinovirus species were characterized by PCR.

**Results:**

Rhinovirus was detected in 42.22% samples (236/559), RV-A was detected in 10.17% (24/236) of the cases, RV-B in 16.53% (39/236), and RV-C in 73.31% (173/236). The age group with the highest number of cases was the 0–5 months group with 45.97%, followed by the 1–5 years group with 25.22%. Most of the positive RV cases, i.e., 86.44% (204/236), were hospitalized. The most common signs and symptoms found in patients who tested positive for RV were cough (72.88%), fever (68.64%), rhinorrhea (68.22%), and respiratory distress (61.44%). Infection with RV-A was associated with wheezing (p = 0.02). Furthermore, RV-C was related to cough (p = 0.01), wheezing (p = 0.002), and conjunctival injection (p = 0.03). A peak in RV-C cases was found in March (32 cases in 2010); June (18 cases in 2009 and 12 cases in 2010), which corresponds to the fall season in Peru; and also November (17 cases in 2009 and 4 cases in 2010), which corresponds to spring. RV-A and RV-B cases were constant throughout the year.

**Conclusion:**

In conclusion, we found a high prevalence of rhinovirus C infection among pediatric patients with acute respiratory infections in Lima, Peru. This viral infection was more common in children between 0 to 5 months old, and was associated with cough, wheezing, and conjunctival injection. Epidemiological surveillance of this virus should be strengthened/encouraged in Peru to determine its real impact on respiratory infections.

## Introduction

Acute respiratory infections (ARIs) are one of the major causes of morbidity and mortality worldwide. They can be classified into upper respiratory tract infections (URTIs), which include pharyngitis, laryngitis, and sinusitis; and lower respiratory tract infections (LRTI), which include bronchiolitis, bronchitis, and pneumonia [1]. In particular, lower respiratory tract infections rank fourth in the leading causes of death in all age groups according to the World Health Organization (WHO) [2]. Moreover, respiratory tract infections remain as the leading cause of death in children under 5 years old, affecting lower and middle-income countries predominantly [3, 4]. Viral pathogens are the main causes of ARIs, accounting for more than 80% of the cases [5]. The most important viruses include human rhinovirus (RV), influenza A and B (FLuA, FluB), respiratory syncytial virus (RSV), adenovirus, parainfluenza virus (PIV), and human metapneumovirus (hMPV), among others. Bacterial pathogens, such as *Streptococcus pneumoniae*, *Haemophilus influenzae*, *Staphylococcus aureus*, and *Moraxella catarrhalis*, among others [5–7], are reported less frequently.

Human rhinovirus (RV) has been traditionally associated with URTIs, which represent the major cause of common cold worldwide [8, 9]. Nonetheless, as recent advances in molecular diagnostic techniques are conducted, this virus has been identified as a lower respiratory tract pathogen, particularly in infants, immunocompromised patients, and the elderly [9]. Recent evidence indicates that RV may be a significant cause of pneumonia, bronchiolitis and asthma exacerbation in children [10, 11]. For example, RV has been reported with a prevalence that ranges from 20% to 40% in children with bronchiolitis, just behind respiratory syncytial virus (RSV) [10–12]. There has also been found an increasing prevalence of RV in children with respiratory diseases that were admitted to pediatric intensive care units [13].

During last years, more than 170 serotypes of human rhinovirus have been discovered, which have been grouped into 3 species: rhinovirus A, B and C [8]. Patients with RV infection often develop only a mild respiratory disease, with cough, fever and wheezing as the most common symptoms [12]. However, each species of RV possesses a unique pathophysiology and may be related to different clinical presentations [14, 15]. For example, RV-A is more common in patients with bronchiolitis and pneumonia, while RV-C is associated with bronchiolitis and asthma exacerbation. On the other hand, RV-B has been related to mild respiratory disease or has an asymptomatic presentation [15–17]. Finally, the most commonly reported species in children with respiratory infections are types A and C [17–19].

Neither epidemiology of human rhinovirus nor its role in respiratory infections in children in Peru are known yet. Therefore, the aim of this study was to describe the prevalence of rhinovirus and to identify the circulating species in nasopharyngeal swabs samples taken from children with acute respiratory infections.

## Materials and methods

### Study design, patients and samples

The study consists of a secondary analysis of nasopharyngeal swab samples from a previous cross-sectional study in children younger than 17 years old with clinical diagnosis of ARI from the “Hospital Nacional Cayetano Heredia” between May 2009 and December 2010. The original study recruited more than 1,000 inpatients with ARI, 559 samples of which were included and analyzed in the current study. The partial results of this study were published previously and showed the analysis of 767 patients [20].

Inclusion criteria were pediatric patients with a clinical diagnosis of acute respiratory infection, whose parent or guardian signed the informed consent. Exclusion criteria were patients with a history of immunodeficiency, as well as samples in an inadequate state of preservation.

**Fig 1:**
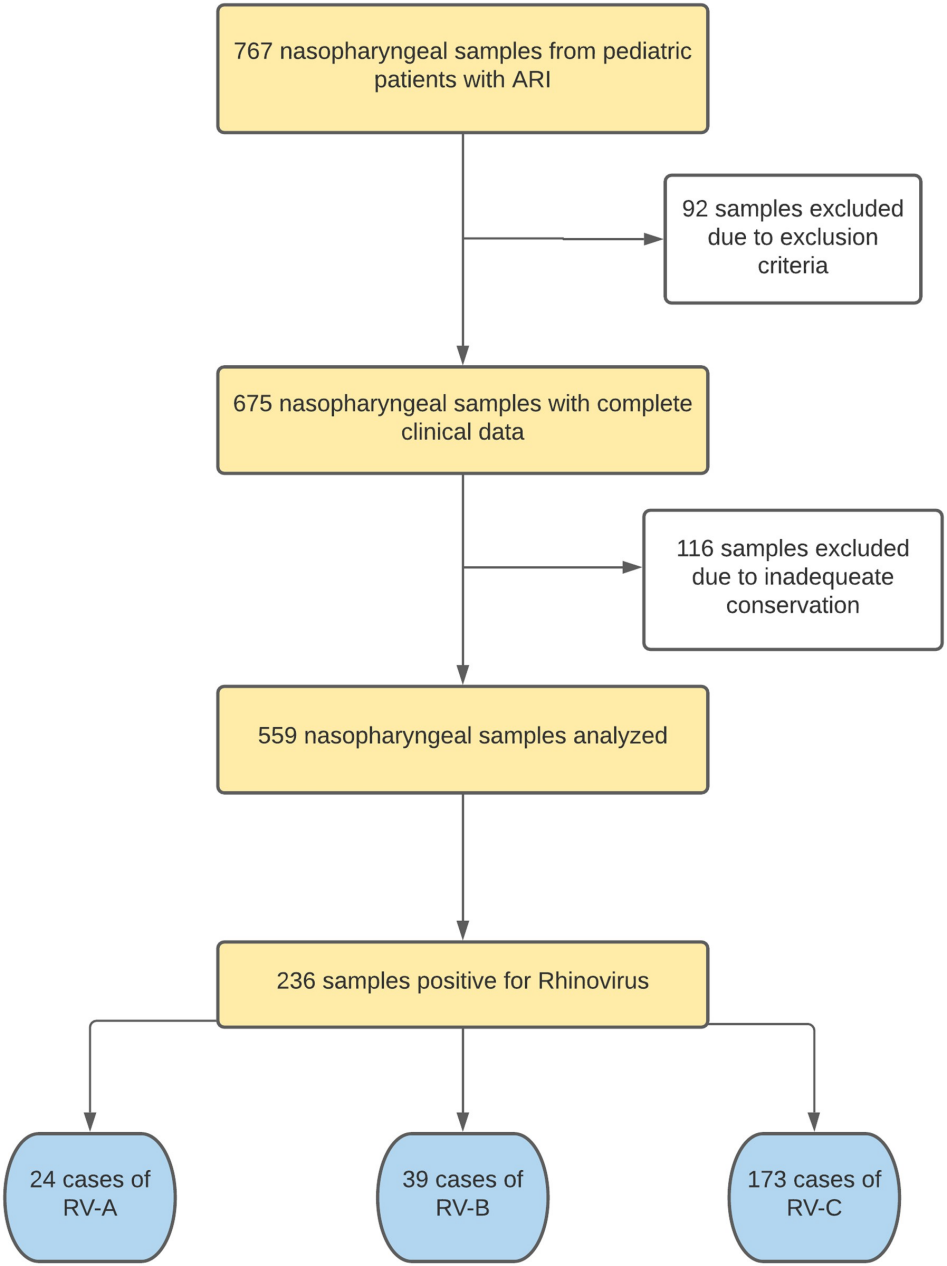
Flowchart of the study design and procedures.

Epidemiological and clinical characteristics were registered on a database with the following information: age, symptoms (fever, rhinorrhea, cough, respiratory distress, sore throat, wheezing, malaise, pharyngeal congestion, expectoration, vomit, and diarrhea, among others), duration of symptoms, and clinical outcomes. Fig 1 shows the procedures, design, and results of the current study. A total of 767 nasopharyngeal samples were collected from pediatric patients with ARI, 559 of which were included according to the inclusion and exclusion criteria.

We used two nasopharyngeal samples by inserting one swab on both nasal cavities parallel to the palate (Mini-Tip Culture Direct, Becton-Dickinson Microbiology System, MD) and a second swab in the pharynx and posterior tonsil (Viral Culturette, Becton-Dickinson Microbiology Systems, MD). Samples collected were then put into the same tube that contained the viral transport media (an essential solution buffered with NaHCO3 and supplemented with fetal bovine serum at 2%, penicillin and streptomycin 100U/ml, amphotericin B 20mg/mL, and neomycin 40mg/mL). Samples from the original study were stored at -80°C in the molecular biology laboratory at Instituto de Investigación Nutricional—Universidad Peruana de Ciencias Aplicadas (Lima, Peru).

### Nucleic acid extraction (RNA)

RNA extraction was conducted from 200μL of nasopharyngeal swab samples. RNA was extracted with the High Pure RNA Isolation Kit (Roche Applied Science, Mannheim, Germany) according to the manufacturer’s instructions.

### cDNA synthesis

The cDNA Transcriptor High Fidelity synthesis kit (Roche Applied Science, Mannheim, Germany) was used according to the manufacturer’s instructions. Regarding the cDNA synthesis, we used 2.5 μL of RNA, 1 μL of random primer, and 3 μL of water; samples were then incubated for 10 minutes at 65 ºC. Afterwards, 2 μL of buffer, 0.25 μL of RNases inhibitor, 1 μL of dNTPs, and 0.25 μL of transcriptor reverse (20U/μL) were added, and samples were incubated at 55 ºC for 30 minutes, followed by an inactivation cycle at 85 ºC for 5 minutes.

### Conventional PCR to amplify rhinovirus, RV-A and RV-B

The detection of rhinovirus and differentiation of rhinovirus species A and B were performed using the primers that were previously described by Jin et al. [21] and Lu et al. [22], as shown in S1 Table. PCR assay was conducted using primers at a concentration of 20 picomoles; each reaction (20 μl) contained 2 μl of cDNA solution, 1 μl of each primer, and 16 μl Master Mix (FastStart Taq DNA Polymerase, reaction buffer, MgCl_2_, deoxynucleoside triphosphates, and water) (Roche Diagnostic, GmbH, Mannheim, Germany). Amplifications were initiated with an incubation at 95 ºC for 10 minutes, followed by 45 cycles of 95 ºC for 30 seconds, 55 ºC for 45 seconds, and 62 ºC for 60 seconds; after these cycles, a final extension at 72 ºC was conducted for 10 minutes. The amplified products were observed in 1.5% TBE agarose gel with 3 μg/ml of ethidium bromide and were photographed under ultraviolet illumination (Transilluminator UV KODAC LOGIC 1500, New Haven, USA). The amplified products were recovered from the gel, purified (SpinPrepTM Gel DNA Kit, San Diego, USA) and sent for commercial sequencing service (Macrogen, Seoul, Korea) to confirm the results.

### Real-time PCR amplify rhinovirus C (RV-C)

PCR assay was carried out using TaqMan probe with FAM-TAMRA fluorescence at a concentration of 50 μM and primers at a concentration of 20 picomoles. Each reaction (20 μl) consisted of 2 μl of cDNA solution, 1 μl of each primer, 1 μl of TaqMan probe, and 15 μl Master Mix (FastStart Taq DNA Polymerase, reaction buffer, MgCl_2_, deoxynucleoside triphosphates, and water) (Roche Diagnostic, GmbH, Mannheim, Germany). The PCR conditions for RV-C were 95°C for 10 minutes and 45 cycles of 15 seconds at 95°C, 30 seconds at 52°C, and 30 seconds at 72°C. The procedure was carried out in Light Cycler 2.0 and the results were analyzed with the Light Cycler version 4.1 software (Roche Diagnostics, Mannheim, Germany). The primers and probe used (S1 Table) were described previously by Sikazwe et al. [23].

### PCR to amplify influenza virus, parainfluenza virus and respiratory syncytial virus

The detection of influenza A, B, and C virus, respiratory syncytial virus (RSV-A and RSV-B), and parainfluenza virus (PIV-1, PIV-2, PIV-3, and PIV-4) was conducted using the specific primers that Coiras et al. [24] described previously. The PCR conditions was as described by del Valle et al. [20]. Finally, the viral etiology was determined when RT-PCR samples were positive for at least one of these viruses.

### Data analysis

Categorical variables were described as frequencies and percentages for each group. The frequency distribution among groups was analyzed using Chi square test and Fisher’s exact test using STATA 16.0 software, and the graphs were created using the GraphPad Prism 9.0.1 software (San Diego, California, USA). Furthermore, the univariate and multivariate analyses were carried out to analyze the clinical signs and symptoms in patients with co-infections (influenza, RSV, PIV) compared to monoinfections by rhinovirus species, for which the multinominal logistic regression was used. Variables with a p-value <0.2 in the univariate analysis were included in the multivariate analysis. A p-value <0.05 was considered statistically significant.

### Ethics statement

This study was approved by the ethics committee from the National Hospital Cayetano Heredia (Ethical Application No. 021–09) and the Instituto de Investigación Nutricional (Ethical Application No. 279-2009/CEI-IIN). The ethical approval was updated in 2020 for the re-analysis of the samples for RV: Universidad Peruana de Ciencias Aplicadas (Ethical Application No. 298-08-20/August-2020) in Lima, Peru. Samples were collected after a parent or guardian signed the informed consent to agree to participate. All procedures were performed under the international ethics guidelines for research in human healthcare issued by the Council for International Organizations of Medical Sciences (CIOMS) and the World Health Organization (WHO).

## Results

The detection of human rhinovirus (RV) and the specific species (A, B and C) was performed through molecular methods. We found that RV was detected in 42.22% samples (236/559), RV-A in 10.17% (24/236) of the cases, RV-B in 16.53% (39/236), and RV-C in 73.31% (173/236).

The demographic characteristics of the patients are described in Table 1. We observed, among RV cases, that 57.20% were male (135/236) and 40.25% (95/236) were female. Regarding age, the group with the highest number of cases was the 0-5-month-old group with 45.97%, followed by the 1–5-year-old group with 25.22%. The group with the least detected cases was 11–17 years old with 3.76% of cases. Most of the RV positive cases, 86.44% (204/236), were hospitalized. As per the rhinovirus species, the hospitalization rates were observed as follows: 87.50% (21/24) for RV-A, 79.49% (31/39) for RV-B, and 87.28% (151/173) for RV-C. regarding RV species, there were no significant differences when comparing age and gender.

**Table 1:** Demographic characteristics of pediatric patients with clinical diagnosis of ARI and rhinovirus infection.

	Total cases N = 559 (%)	Rhinovirus N = 236 (%)	Rhinovirus A N = 24 (%)	P-value	Rhinovirus B N = 39 (%)	P-value	Rhinovirus C N = 173 (%)	P-value
**Age**								
**0–5 months**	257 (45.97)	115 (48.73)	14 (58.33)		23 (58.97)		78 (45.09)	
**6–11 months**	102 (18.25)	49 (20.76)	6 (25.00)		6 (15.38)		37 (21.39)	
**1–5 years**	141 (25.22)	54 (22.88)	1 (4.17)	0.070(+)	9 (23.08)	0.417(+)	44 (25.43)	0.505(*)
**6–10 years**	32 (5.72)	10 (4.24)	2 (8.33)		0 (0.00)		8 (4.62)	
**11–17 years**	21 (3.76)	6 (2.54)	1 (4.17)		1 (2.56)		4 (2.31)	
**NA**	6 (1.07)	2 (0.85)	0 (0.00)		0 (0.00)		2 (1.16)	
**Gender**								
**Female**	228 (40.79)	95 (40.25)	10 (41.67)		12 (30.77)		73 (42.2)	
**Male**	318 (56.89)	135 (57.20)	13 (54.17)	0.864(*)	27 (69.23)	0.149(*)	95 (54.91)	0.593(*)
**NA**	13 (2.33)	6 (2.54)	1 (4.17)		0 (0.00)		5 (2.89)	
**Hospitalization**								
**Yes**	467 (83.54)	204 (86.44)	21 (87.5)	0.781(+)	32 (82.05)	0.795(*)	151 (87.28)	0.110(*)
**No**	92 (16.46)	32 (13.56)	3 (12.50)		7 (17.95)		22 (12.72)	

(*) Chi square test

(^+^) Exact Fisher test

NA: not available

As shown in Table 2, the most common signs and symptoms found in patients with a positive diagnosis of rhinovirus were cough (72.88%), fever (68.64%), rhinorrhea (68.22%), and respiratory distress (61.44%). When comparing the distribution of symptoms according to the rhinovirus species, the results indicated that infection with RV-A was associated with wheezing (p = 0.02). Moreover, RV-C was related to cough (p = 0.01), wheezing (p = 0.002), and conjunctival injection (p = 0.03).

**Table 2:** Clinical characteristics of pediatric patients with clinical diagnosis of ARI and rhinovirus infection.

Signs and symptoms	Total cases N = 559 (%)	Rhinovirus N = 236 (%)	Rhinovirus A N = 24 (%)	Rhinovirus B N = 39 (%)	Rhinovirus C N = 173 (%)
**Cough**	389 (69.59)	172 (72.88)	16 (66.67)	23 (58.97)	**133 (76.88)** *
**Fever**	379 (67.80)	162 (68.64)	17 (70.83)	27 (69.23)	118 (68.21)
**Rhinorrhea**	374 (66.90)	161 (68.22)	17 (70.83)	23 (58.97)	121 (69.94)
**Respiratory distress**	317 (56.71)	145 (61.44)	14 (58.33)	24 (61.54)	107 (61.85)
**Wheezing**	203 (36.31)	104 (44.07)	**14 (58.33)** *	11 (28.21)	**79 (45.66)** *
**Malaise**	142 (25.40)	70 (29.66)	8 (33.33)	12 (30.77)	50 (28.90)
**Expectoration**	138 (24.69)	61 (25.85)	7 (29.17)	7 (17.95)	47 (27.17)
**Pharyngeal congestion**	135 (24.15)	54 (22.88)	6 (25.00)	6 (15.38)	42 (24.28)
**Sore throat**	73 (13.06)	35 (14.83)	5 (20.83)	6 (15.38)	24 (13.87)
**Vomiting**	70 (12.52)	36 (15.25)	5 (20.83)	9 (23.08)	22 (12.72)
**Diarrhea**	57 (10.20)	27 (11.44)	3 (12.50)	2 (5.13)	22 (12.72)
**Asthenia**	47 (8.41)	14 (5.93)	2 (8.33)	1 (2.56)	11 (6.36)
**Headache**	30 (5.37)	9 (3.81)	2 (8.33)	2 (5.13)	5 (2.89)
**Abdominal pain**	26 (4.65)	12 (5.08)	1 (4.17)	2 (5.13)	9 (5.20)
**Conjunctival injection**	26 (4.65)	15 (6.36)	0 (0.00)	2 (5.13)	**13 (7.51)** *
**Shock**	11 (1.97)	5 (2.12)	0 (0.00)	2 (5.13)	3 (1.73)
**Adenopathy**	10 (1.79)	6 (3.81)	2 (8.33)	1 (2.56)	3(1.73)
**Otalgia**	9 (1.61)	2 (0.85)	0 (0.00)	0 (0.00)	2(1.16)
**Myalgias**	8 (1.43)	2 (0.85)	1 (4.17)	0 (0.00)	1(0.58)
**Consciousness alteration**	7 (1.25)	4 (1.69)	0 (0.00)	1 (2.56)	3(1.73)
**Multiorgan failure**	6 (1.07)	4 (1.69)	0 (0.00)	2 (5.13)	2(1.16)
**Rash**	6 (1.07)	1 (0.42)	0 (0.00)	0 (0.00)	1(0.58)
**Photophobia**	4 (0.72)	0 (0.00)	0 (0.00)	0 (0.00)	0 (0.00)

* Chi square test, p-value <0.05

We described the detection of other common viruses in these patients. In general, RSV-A was the most prevalent respiratory virus in all RV positive samples, as well as in RV negative patients. We could highlight that patients with RV-C infection had a higher detection of RSV-A (14.45%). Other viruses, such as influenza and parainfluenza, were less frequently detected in all groups (Table 3).

**Table 3:** Co-infections between rhinovirus and other respiratory viruses.

		Rhinovirus A N = 24 (%)	Rhinovirus B N = 39 (%)	Rhinovirus C N = 173 (%)	Rhinovirus Negative N = 323 (%)
**Influenza**	Influenza A	1 (4.17)	2 (5.13)	3 (1.73)	10 (3.09)
	Influenza B	0 (0.00)	0 (0.00)	1 (0.58)	0 (0.00)
	Influenza C	0 (0.00)	0 (0.00)	1 (0.58)	0 (0.00)
**RSV**	RSV-A	1 (4.17)	3 (7.69)	25 (14.45)	40 (12.38)
	RSV-B	0 (0.00)	0 (0.00)	2 (1.16)	1 (0.31)
**Parainfluenza**	Parainfluenza 1	1 (4.17)	0 (0.00)	6 (3.47)	11 (3.41)
	Parainfluenza 2	1 (4.17)	0 (0.00)	1 (0.58)	8 (2.48)
	Parainfluenza 3	1 (4.17)	0 (0.00)	0 (0.00)	6 (1.86)
	Parainfluenza 4	0 (0.00)	0 (0.00)	2 (1.16)	6 (1.86)
**Monoinfection**		20 (83.33)	34 (87.18)	135 (78.03)	-

Table 4 shows the univariate and multivariate analysis of the signs and symptoms among monoinfections and co-infections. In the univariate analysis, patients co-infected with RV-B/influenza were more likely to present headaches and abdominal pain compared to RV-B monoinfections (p<0.05). Likewise, patients co-infected with RV-C/RSV were more likely to present cough and asthenia, compared to RV-C monoinfections. In the case of RV-C/PIV co-infection, patients were more likely to present abdominal pain. These associations remained statistically significant in the multivariate analysis.

**Table 4:** Clinical characteristics of pediatric patients with rhinovirus infections and co-infections.

Symptoms	Rhinovirus A				Rhinovirus B				Rhinovirus C			
	Monoinfection N = 20 (%)	Flu N = 1 (%)	RSV N = 1 (%)	PIV N = 3 (%)	Monoinfection N = 34 (%)	Flu N = 2 (%)	RSV N = 3 (%)	PIV N = 0 (%)	Monoinfection N = 135 (%)	Flu N = 5 (%)	RSV N = 27 (%)	PIV N = 9 (%)
**Cough**	12 (60.00)	1 (100.00)	1 (100.00)	3 (100.00)	18 (52.94)	2 (100.00)	3 (100.00)	0 (0.00)	98 (72.59)	4 (80.00)	25 (92.60)*	8 (88.89)
**Fever**	14 (70.00)	1 (100.00)	1 (100.00)	2 (66.67)	22 (64.71)	2 (100.00)	3 (100.00)	0 (0.00)	95 (69.85)	4 (80.00)	17 (62.90)	4 (44.44)
**Rhinorrhea**	14 (70.00)	0 (0.00)	1 (100.00)	3 (100.00)	18 (52.94)	2 (100.00)	3 (100.00)	0 (0.00)	93 (68.14)	2 (40.00)	22 (81.50)	6 (66.67)
**Respiratory distress**	10 (50.00)	1 (100.00)	1 (100.00)	3 (100.00)	20 (58.82)	1 (50.00)	3 (100.00)	0 (0.00)	82 (60.74)	2 (40.00)	16 (59.30)	8 (88.89)
**Wheezing**	11 (55.00)	0 (0.00)	1 (100.00)	3 (100.00)	9 (26.47)	0 (0.00)	2 (66.67)	0 (0.00)	60 (44.44)	1 (20.00)	15 (55.60)	4 (44.44)
**Malaise**	5 (25.00)	1 (100.00)	1 (100.00)	2 (66.67)	11 (32.35)	1 (50.00)	1 (33.33)	0 (0.00)	41 (30.37)	1 (20.00)	6 (22.20)	3 (33.33)
**Expectoration**	4 (20.00)	1 (100.00)	1 (100.00)	2 (66.67)	5 (14.71)	1 (50.00)	1 (33.33)	0 (0.00)	38 (28.15)	0 (0.00)	8 (29.60)	1 (11.11)
**Pharyngeal congestion**	4 (20.00)	0 (0.00)	1 (100.00)	2 (66.67)	3 (8.82)	1 (50.00)	1 (33.33)*	0 (0.00)	32 (23.70)	0 (0.00)	9 (33.30)	1 (11.11)
**Sore throat**	3 (15.00)	0 (0.00)	1 (100.00)	2 (66.67)	4 (11.76)	2 (100.00)	0 (0.00)	0 (0.00)	17 (12.59)	0 (0.00)	6 (22.20)	1 (11.11)
**Vomiting**	4 (20.00)	0 (0.00)	1 (100.00)	1 (33.33)	7 (20.59)	1 (50.00)	1 (33.33)	0 (0.00)	14 (10.37)	0 (0.00)	6 (22.20)	2 (22.22)
**Diarrhea**	2 (10.00)	0 (0.00)	1 (100.00)	1 (33.33)	1 (2.94)	0 (0.00)	0 (0.00)	0 (0.00)	15 (11.11)	0 (0.00)	5 (18.50)	2 (22.22)
**Asthenia**	1 (5.00)	0 (0.00)	0 (0.00)	1 (33.33)	1 (2.94)	0 (0.00)	0 (0.00)	0 (0.00)	5 (3.70)	1 (20.00)	6 (22.20)*	0 (0.00)
**Headache**	2 (10.00)	0 (0.00)	0 (0.00)	0 (0.00)	1 (2.94)	1 (50.00)*	0 (0.00)	0 (0.00)	5 (3.70)	0 (0.00)	0 (0.00)	0 (0.00)
**Abdominal pain**	1 (50.00)	0 (0.00)	0 (0.00)	0 (0.00)	1 (2.94)	1 (50.00)*	0 (0.00)	0 (0.00)	6 (4.44)	0 (0.00)	1 (3.70)	2 (22.22)*
**Conjunctival injection**	0 (0.00)	0 (0.00)	0 (0.00)	0 (0.00)	1 (2.94)	0 (0.00)	1 (33.33)	0 (0.00)	9 (6.67)	0 (0.00)	3 (11.10)	1 (11.11)
**Shock**	0 (0.00)	0 (0.00)	0 (0.00)	0 (0.00)	2 (5.88)	0 (0.00)	0 (0.00)	0 (0.00)	2 (1.48)	0 (0.00)	1 (3.70)	0 (0.00)
**Adenopathy**	0 (0.00)	0 (0.00)	1 (100.00)	0 (0.00)	1 (2.94)	0 (0.00)	0 (0.00)	0 (0.00)	2 (1.48)	0 (0.00)	1 (3.70)	0 (0.00)
**Otalgia**	0 (0.00)	0 (0.00)	0 (0.00)	0 (0.00)	0 (0.00)	0 (0.00)	0 (0.00)	0 (0.00)	2 (1.48)	0 (0.00)	0 (0.00)	0 (0.00)
**Myalgias**	0 (0.00)	0 (0.00)	0 (0.00)	2 (66.67)	0 (0.00)	0 (0.00)	0 (0.00)	0 (0.00)	1 (0.74)	0 (0.00)	0 (0.00)	0 (0.00)
**Consciousness alteration**	0 (0.00)	0 (0.00)	0 (0.00)	0 (0.00)	1(2.94)	0 (0.00)	0 (0.00)	0 (0.00)	3 (2.22)	0 (0.00)	0 (0.00)	0 (0.00)
**Multiorgan failure**	0 (0.00)	0 (0.00)	0 (0.00)	0 (0.00)	2 (5.88)	0 (0.00)	0 (0.00)	0 (0.00)	2 (1.48)	0 (0.00)	0 (0.00)	0 (0.00)
**Rash**	0 (0.00)	0 (0.00)	0 (0.00)	0 (0.00)	0 (0.00)	0 (0.00)	0 (0.00)	0 (0.00)	1 (0.74)	0 (0.00)	0 (0.00)	0 (0.00)

* Univariate analysis using polychotomous logistical model (monoinfection vs coinfection), P-value < 0.05

^ɸ^ Multivariate analysis using polychotomous logistical model (monoinfection vs coinfection), P-value < 0.05

**Fig 2:**
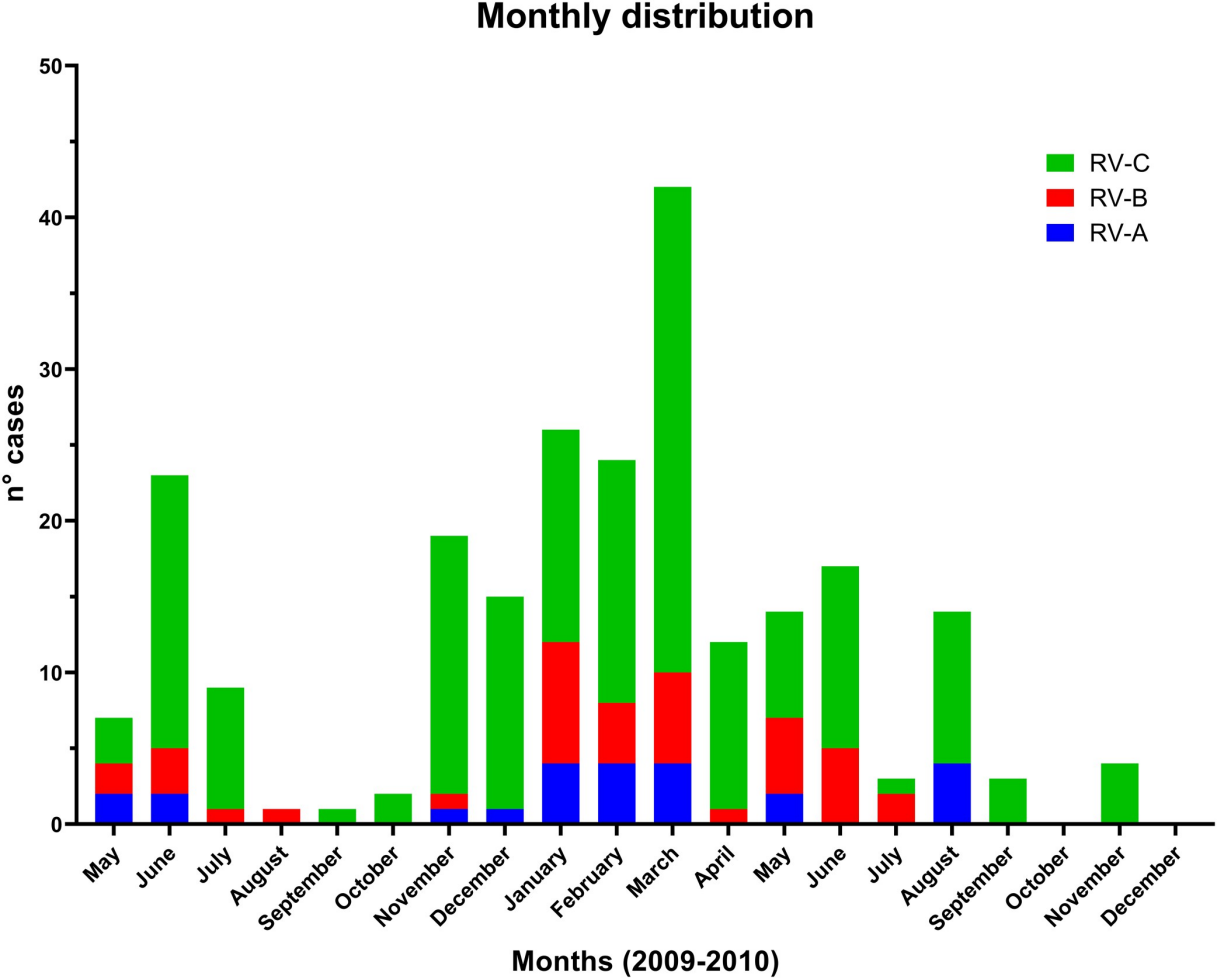
Confirmed cases of rhinovirus infection per month during 2009–2010.

Finally, we analyzed the monthly distribution of RV cases to better describe the epidemiology of this infection. Fig 2 shows the monthly distribution of RV cases and the specific species found throughout the year. A peak in cases of RV-C was found in March (32 cases in 2010), June (18 cases in 2009 and 12 cases in 2010) and November (17 cases in 2009 and 4 cases in 2010). RV-A and RV-B cases were constant throughout the year. The months with the least cases for all RV species were September and October in both years. Although, we observed some peaks in specific months, the survey duration was not long enough to have a definite seasonality pattern of RV infections.

## Discussion

In the last decades, significant advances have been made in the epidemiological surveillance of RV due to the greater availability of molecular tests, particularly in the identification of the virus species and their relationship with different diseases. Although this virus has traditionally been related to common cold and upper respiratory tract infections, recent evidence indicates that this pathogen is implicated in lower respiratory tract infections such as bronchiolitis and asthma exacerbations [11, 15]. Making a timely etiological diagnosis leads to a better patient follow-up and targeted supportive treatment; therefore, we performed a study on rhinovirus epidemiology, as well as its most common species implicated in ARI infections in children in Peru. In the current study, we have found that 42.22% of children with ARI were positive for RV, 10.17% of which were diagnosed with RV-A, 16.53% with RV-B, and 73.31% with RV-C. RV Infection was more prevalent in patients between 0 to 5 months old, followed by patients between 1 to 5 years old and 6 to 11 months old.

Previous studies have evaluated the prevalence of RV and its most common species in pediatric patients with ARI and have obtained varying results depending on the geographical area and season. For example, a study conducted by Howard et al. [17] evaluated the most common species of RV from pediatric patients with respiratory infections in Cajamarca, Peru. They reported a prevalence of 50.2% of RV-A, 9.7% of RV-B, and 40.1% of RV-C, among 207 positive RV samples. Another study performed in Brazil [16] found that, among 630 children with ARI, the RV infection was detected in 18.7%, 73% of which corresponded to RV-A and 27% to RV-C. Wildenbeest et al. [12] reported that the predominant species of RV in young infants from the Netherlands were RV-A with 59%, followed by RV-C with 32% and RV-B with 9%; moreover, RV-B infection was associated with asymptomatic presentation.

Likewise, Garcia et al. [25] carried out one of the largest studies on RV epidemiology in Latin America. They studied 3375 young patients with influenza-like illness, 16% of which were detected to have RV. Among these cases, RV-C accounted for 38% of all RV infections. Another important study that was performed by Chen et al. [26] studied 84 cases of RV, among 160 samples from children and adults. Similar to our study, HRV-C was detected more frequently among children (13/22, 59%) than adults (6/62, 10%). In our study, we demonstrated that the predominating RV species in pediatric patients that circulated in this study was RV-C with 73.3% of the total infections. This highlights the need to strengthen the epidemiological surveillance on respiratory diseases, as expansion of this novel species may be ongoing. There are heterogeneous results on rhinovirus epidemiology, which may be explained by differences in geographic areas, study periods, study groups, and diagnostic method used.

We assessed the frequency of different symptoms and clinical presentation in relation to RV infection and specific species. This current study found that the most frequent symptoms in RV infection were cough, fever, rhinorrhea, and respiratory distress. Similarly, when evaluating specific RV species, we found that RV-A infection was associated with wheezing, while RV-C infection was related to cough, wheezing, and conjunctival injection. A variety of studies have made evident those classic symptoms in RV infections, which consist of rhinorrhea, sore throat, cough, fever, and wheezing, among others [8, 14]. Furthermore, some studies also suggest that the infection by specific RV species may be associated with certain clinical presentation. For example, RV-A infection has been related to mild disease, with no characteristic symptoms [18, 19]; nonetheless, we found that wheezing can be related to this infection. RV-B infections were the second most common species in our study; however, no characteristic signs and symptoms were identified in this species. In fact, infections by this virus species are frequently asymptomatic or present with mild disease [12]. Lee et al.’s study [27] reported that RV-B infection was less likely to produce moderate to severe respiratory illness compared to species A and C. Infections by RV-C were the most common in our study and were related to greater frequency of wheezing, cough, and conjunctival injection. There is increasing evidence that indicates that this species may be the most important due to its rapid expansion and its impact in morbidity. Previous studies indicate that RV-C is related to lower respiratory tract infections, greater respiratory distress, and the need for supplementary oxygen [15–17]. Moreover, infections by this species have been increasingly associated with asthma exacerbations, as well as persisting wheezing episodes [11]. Particular attention should be given to this pathogen, as our results indicate that more than 70% of the cases corresponded to RV-C. Further epidemiological and longitudinal analysis should be performed to estimate the impact of this infection in long-term wheezing, sensitization, and asthma.

One important aspect of our study was that some patients were co-infected with other respiratory viruses, such as influenza, RSV, and parainfluenza, which could have a role in the clinical picture. In contrast, previous studies show that co-detection of RV and other respiratory viruses did not increase the severity of the disease [25, 28]. We report that some clinical signs and symptoms were associated with co-infections in comparison with monoinfections. For example, abdominal pain was associated with RV-B/flu and RV-C/PIV. Headaches were more likely in patients with RV/flu. Lastly, patients with RV-C/RSV were more likely to present cough and asthenia. These findings are similar to those Papadopoulos et al. [29] reported, which clinical picture was different when co-infections between RV and other respiratory viruses were present.

Finally, the monthly distribution of RV infections and the specific species was evaluated. We observed a constant distribution throughout the year, with peaks of infections in March and June, which correspond to the fall season in our country. This season is usually a dry season, with warm temperatures that oscillate between 19 C° to 24 C°. Nevertheless, data on RV seasonality is heterogeneous and varies according to the area of study. For example, a large study in Latin America [25] found that RV-A and RV-B cases were constant throughout the year and were not related to rainy or high temperature seasons. However, RV-C prevailed in countries located north of the equator over the months of September 2010 to January 2011, and the opposite took place in regions located south of the equator, where the detection of HRV-C increased during April 2011 to July 2011 [25]. A study on Mozambique [18] found that cases were constant throughout the year with a peak during the warm and wet months. Another study in Brazil [16] found a peak in cases during the months of May, June and July, which correspond to the local rainy season. The variable monthly distribution of this virus could be explained due to different local weather conditions such as humidity and different social factors.

## Limitations

One of the main limitations of this study was that samples were collected 10 years ago. This study was performed retrospectively from a secondary analysis of nasopharyngeal swab samples previously collected; therefore, some clinical data of interest was not available. Information of comorbidities, such as asthma or previous wheezing episodes, could be important variables to study. Another limitation is that the present study was performed in a single reference center in Lima, Peru; therefore, the results may not be extrapolated to other settings with different epidemiology of respiratory pathogens. Similarly, as the study was performed at a referral hospital, most of the cases were moderate/severe, which explains the high rates of hospitalization.

In conclusion, we found a high prevalence of rhinovirus C infection among pediatric patients with acute respiratory infections in Lima, Peru. Infection by this virus was more common in children between 0 to 5 months old, and was associated with cough, wheezing and conjunctival injection. The monthly distribution shows an increase in cases during the months of March and June; however, this pathogen may be circulating throughout the year. Epidemiological surveillance of this virus should be strengthened and encouraged in Peru, and a longitudinal analysis should be also performed to determine the role of this pathogen in asthma exacerbations.

## Supporting information

S1 Table Primers and probes to amplify rhinovirus genus and species. (DOCX)

## References

[1] Thomas M, Koutsothanasis G, Bomar P. Upper respiratory tract infection. StatPearls [Internet]. 2020 [cited 22 June 2021]. https://www.ncbi.nlm.nih.gov/books/NBK532961/

[2] Global health estimates: Leading causes of death [Internet]. WHO. 2021 [cited 22 June 2021]. https://www.who.int/data/gho/data/themes/mortality-and-global-health-estimates/ghe-leading-causes-of-death

[3] Avendaño CaRV-Ajal L, Perret Pérez C. Epidemiology of Respiratory Infections. *Pediatric Respiratory Diseases*. 2020;263–272. Published 2020 Feb 1.

[4] Strong KL, Pedersen J, White Johansson E, Cao B, Diaz T, Guthold R, et al. Patterns and trends in causes of child and adolescent mortality 2000–2016: setting the scene for child health redesign. BMJ Glob Health. 2021 Mar;6(3):e004760. doi: 10.1136/bmjgh-2020-004760 33731440 PMC7978083

[5] Mahony JB. Detection of respiratory viruses by molecular methods. *Clin Microbiol Rev*. 2008;21(4):716–747. doi: 10.1128/CMR.00037-07 18854489 PMC2570148

[6] Liu P, Xu M, He L, Su L, Wang A, Fu P, et al. Epidemiology of Respiratory Pathogens in Children with Lower Respiratory Tract Infections in Shanghai, China, from 2013 to 2015. Jpn J Infect Dis. 2018 Jan 23;71(1):39–44. doi: 10.7883/yoken.JJID.2017.323 29279451

[7] Liu WK, Liu Q, Chen DH, Liang HX, Chen XK, Chen MX, et al. Epidemiology of acute respiratory infections in children in Guangzhou: a three-year study. PLoS One. 2014 May 5;9(5):e96674. doi: 10.1371/journal.pone.0096674 24797911 PMC4010508

[8] Jacobs SE, Lamson DM, St George K, Walsh TJ. Human rhinoviruses. Clin Microbiol Rev. 2013 Jan;26(1):135–62. doi: 10.1128/CMR.00077-12 23297263 PMC3553670

[9] Lau SK, Yip CC, Woo PC, Yuen KY. Human rhinovirus C: a newly discovered human rhinovirus species. Emerg Health Threats J. 2010;3:e2. doi: 10.3134/ehtj.10.002 22460392 PMC3167658

[10] Bizot E, Bousquet A, Charpié M, Coquelin F, Lefevre S, Le Lorier J, et al. Rhinovirus: A Narrative Review on Its Genetic Characteristics, Pediatric Clinical Presentations, and Pathogenesis. Front Pediatr. 2021 Mar 22;9:643219. doi: 10.3389/fped.2021.643219 33829004 PMC8019700

[11] Hasegawa K, Mansbach JM, Bochkov YA, Gern JE, Piedra PA, Bauer CS, et al. Association of Rhinovirus C Bronchiolitis and Immunoglobulin E Sensitization During Infancy With Development of Recurrent Wheeze. JAMA Pediatr. 2019 Jun 1;173(6):544–552. doi: 10.1001/jamapediatrics.2019.0384 30933255 PMC6547078

[12] Wildenbeest JG, van der Schee MP, Hashimoto S, Benschop KS, Minnaar RP, Sprikkelman AB, et al. Prevalence of rhinoviruses in young children of an unselected birth cohort from the Netherlands. Clin Microbiol Infect. 2016 Aug;22(8):736.e9–736.e15. doi: 10.1016/j.cmi.2016.05.022 27265373 PMC7128250

[13] Cox DW, Khoo SK, Zhang G, Lindsay K, Keil AD, Knight G, et al. Rhinovirus is the most common virus and rhinovirus-C is the most common species in paediatric intensive care respiratory admissions. Eur Respir J. 2018 Aug 9;52(2):1800207. doi: 10.1183/13993003.00207-2018 29976655 PMC6295450

[14] Vandini S, Biagi C, Fischer M, Lanari M. Impact of Rhinovirus Infections in Children. Viruses. 2019 Jun 5;11(6):521. doi: 10.3390/v11060521 31195744 PMC6632063

[15] Su YT, Lin YT, Yang CC, Tsai SS, Wang JY, Huang YL, et al. High correlation between human rhinovirus type C and children with asthma exacerbations in Taiwan. J Microbiol Immunol Infect. 2020 Aug;53(4):561–568. doi: 10.1016/j.jmii.2018.12.001 30591259

[16] Fawkner-Corbett DW, Khoo SK, Duarte CM, Bezerra PG, Bochkov YA, Gern JE, et al. Rhinovirus-C detection in children presenting with acute respiratory infection to hospital in Brazil. J Med Virol. 2016 Jan;88(1):58–63. doi: 10.1002/jmv.24300 26100591 PMC4682890

[17] Howard LM, Johnson M, Gil AI, Griffin MR, Edwards KM, Lanata CF, et al. Molecular Epidemiology of Rhinovirus Detections in Young Children. Open Forum Infect Dis. 2016 Jan 13;3(1):ofw001. doi: 10.1093/ofid/ofw001 26900577 PMC4759584

[18] Annamalay AA, Lanaspa M, Khoo SK, et al. Rhinovirus species and clinical features in children hospitalised with pneumonia from Mozambique. Trop Med Int Health. 2016;21(9):1171–1180. doi: 10.1111/tmi.12743 27353724 PMC7169728

[19] Bruning AHL, Thomas XV, van der Linden L. et al. Clinical, virological and epidemiological characteristics of rhinovirus infections in early childhood: A comparison between non-hospitalised and hospitalised children. J Clin Virol. 2015;73:120–126. doi: 10.1016/j.jcv.2015.10.024 26599608 PMC7185867

[20] del Valle Mendoza J, Cornejo-Tapia A, Weilg P, Verne E, Nazario-Fuertes R, Ugarte C, et al. Incidence of respiratory viruses in Peruvian children with acute respiratory infections. J Med Virol. 2015 Jun;87(6):917–24. doi: 10.1002/jmv.24159 25784285 PMC7167149

[21] Jin Y, Yuan XH, Xie ZP, Gao HC, Song JR, Zhang RF, et al. Prevalence and clinical characterization of a newly identified human rhinovirus C species in children with acute respiratory tract infections. J Clin Microbiol. 2009 Sep;47(9):2895–900. doi: 10.1128/JCM.00745-09 19625482 PMC2738104

[22] Lu X, Holloway B, Dare RK, Kuypers J, Yagi S, Williams JV, et al. Real-time reverse transcription-PCR assay for comprehensive detection of human rhinoviruses. J Clin Microbiol. 2008 Feb;46(2):533–9. doi: 10.1128/JCM.01739-07 Epub 2007 Dec 5. 18057136 PMC2238069

[23] Sikazwe CT, Chidlow GR, Imrie A, Smith DW. Reliable quantification of rhinovirus species C using real-time PCR. J Virol Methods. 2016 Sep;235:65–72. doi: 10.1016/j.jviromet.2016.05.014 27216896 PMC7172306

[24] Coiras M, Aguilar J, García M, Casas I. 2004. Simultaneous detection of fourteen respiratory viruses in clinical specimens by two multiplex reverse transcription nested-PCR assays. J Virol. 72:484–495. doi: 10.1002/jmv.20008 14748074 PMC7166637

[25] Garcia J, Espejo V, Nelson M, et al. Human rhinoviruses and enteroviruses in influenza-like illness in Latin America. *Virol J*. 2013;10:305. doi: 10.1186/1743-422X-10-305 24119298 PMC3854537

[26] Chen WJ, Arnold JC, Fairchok MP, et al. Epidemiologic, clinical, and virologic characteristics of human rhinovirus infection among otherwise healthy children and adults: rhinovirus among adults and children. *J Clin Virol*. 2015;64:74–82 doi: 10.1016/j.jcv.2015.01.007 25728083 PMC4347877

[27] Lee WM, Lemanske RF Jr, Evans MD, et al. Human rhinovirus species and season of infection determine illness severity. Am J Respir Crit Care Med 2012; 186:886–91. doi: 10.1164/rccm.201202-0330OC 22923659 PMC3530215

[28] Paula NT, Carneiro BM, Yokosawa J, Freitas GR, Oliveira TF, Costa LF, et al. Human rhinovirus in the lower respiratory tract infections of young children and the possible involvement of a secondary respiratory viral agent. Mem Inst Oswaldo Cruz. 2011 May;106(3):316–21. doi: 10.1590/s0074-02762011000300010 21655819

[29] Papadopoulos NG, Moustaki M, Tsolia M, Bossios A, Astra E, Prezerakou A, et al. Association of rhinovirus infection with increased disease severity in acute bronchiolitis. Am J Respir Crit Care Med. 2002 May 1;165(9):1285–9. doi: 10.1164/rccm.200112-118BC 11991880

