# Supplementary material for: High prevalence and clinical characteristics of respiratory infection by human rhinovirus in children from Lima-Peru during years 2009–2010
Source: PLoS One. 2022 Jul 15;17(7):e0271044. doi: 10.1371/journal.pone.0271044 (PMC9286243; doi:10.1371/journal.pone.0271044)
Supplement: S1 Table — (DOCX) [file pone.0271044.s001.docx]

**Table S1:** Primers and probes to amplify rhinovirus genus and species.

| **Virus** | **Primer** | **Sequence (5´----3´)** | **Tm** |
| --- | --- | --- | --- |
| **Rhinovirus**  Jin Y et al. [21] | **HRV-F** | 5’-GGGACCAACTACTTTGGGTGTCCGTGT-3’ | 62 |
|  | **HRV-R** | 5’- GCATCIGGYARYTTCCACCACCANCC-3’ | 60 |
| **Rhinovirus A (RV-A)**  Lu et al. [22] | **HRVA-F** | 5’- GTACTCTGTTATTCCGGTAACTTTGYAYGCCA-3’ | 70 |
|  | **HRVA-R** | 5’- CCAACATTCTGTCTAGATACYTGDGCVCCCAT-3’ | 71.2 |
| **Rhinovirus B (RV-B)**  Lu et al. [22] | **HRVB-F** | 5’- ACTCTGGTACTATGTACCTTTGTACGCCTGTT-3’ | 71.2 |
|  | **HRVB-R** | 5’- CCACTCTTCTGTGTAGACACYTGDGCDCCCAT-3’ | 73.8 |
| **Rhinovirus C (RV-C)**  Sikazwe et al. [23] | **IrlonS -F** | 5’- GCACTTCTGTTTCCCC-3’ | 50.9 |
|  | **RV-TYPE-C-P3** | **FAM**- CCGCGTGGTGCCC – **TAMRA** | 48 |
|  | **EntA-R** | 5’- GCATTCAGGGGCCGGAG-3’ | 58.9 |
